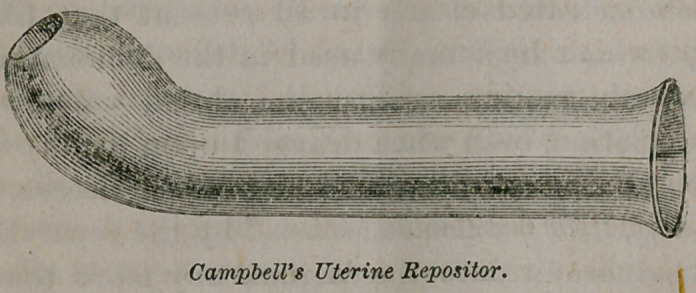# Uterine Displacements Treated by Position, Pneumatic Pressure and Mechanical Appliances

**Published:** 1875-06

**Authors:** H. F. Campbell

**Affiliations:** Augusta, Ga.


ATLANTA

### Medical and Surgical Journal.

Vol. XIII.] JUNE—1875. [No. 3.

### Original Communications.

### THE TREATMENT OF UTERINE DISPLACEMENTS.

Uterine Displacements Treated by Position, Pneumatic Pressure and Mechanical Appliances.

By Prof. H. F. CAMPBELL, M.D., of Augusta, Ga.

By the courtesy of a friend attending the Medical Association of Georgia, we are enabled to give the following abstract of Prof. Campbell's remarks at the recent meeting in Savannah, of which we gave a brief synopsis in our report in the May Journal. This paper was crowded out of that number.—Ed. Jour.

Dr. Henry F. Campbell, of Augusta, Professor of Operative Surgery and Gynæcology in the Medical College of Georgia, was chairman of the Committee on Gynæcology of the Eighth District. He gave a verbal resume of his lengthy report on Uterine Displacements and their Treatment by Position, Pneumatic Pressure and Mechanical Appliance.

Dr. Campbell said : Every sexual abnormality may present two momentous desiderata: first, the health of the patient; secondly, the relief of sterility. Both these ends, it is well known, are materially compromised by uterine displacements. Scarcely any uterine affection can escape complication with some form or degree of malposition; and, on the other hand, no malposition can persistently continue without at least a *liability* to both structural and functional change. He said that though the several affections, comprehended under the general head of uterine displacements, were well understood by the profession, and though they were generally carefully catalogued or tabulated in all works on diseases of women, still there is liability to misunderstanding and confusion in their study, on account of the imperfect and incomplete classification of the several species of malposition. The several forms of the various conditions recognized as "displacements," while they bear sufficient resemblance to each other to admit of class association, still there are *generic differences*, which widely separate them, and which, until they are recognized in the tabulated classifications of the journals and books, must ever lead, and have already led, to much confusion in their discussion. He is aware that any change in the established and long recognized relations of things, even though those relations may be false and inaccurate, is always regarded with disfavor by the profession; but inasmuch as, in this case, the evils of false classification must be felt by every one, and must constantly lead to increasing confusion year after year, he has proposed the following arrangement, the principal feature of which may be said to consist in the *introduction of genera* and the recognition of *generic differences*, by which the vicious and incongruous association of incognate species has been substituted by one which is more congenial and natural. While the nomenclature of uterine displacements is, in general, sufficiently descriptive, new names, in many instances, might be substituted to advantage; still Dr. C. retains the long recognized terms, rather than incur the risk of confusion, which the introduction of new, even though more definite ones, might produce.

TABULAR ARRANGEMENT OF UTERINE DISPLACEMENTS.

DEFINITION.—Conditions of the uterus, more or less affecting the health and fecundity of the subject, in which, whether from *elevation* or *depression;* or from *changed direction;* or from *altered* form, this organ, or any portion of it, has become abnormally related, to either the planes or the axes of its containing cavity, the pelvis, or to the other organs therein contained. They may be recognized under the following several genera:

GENUS 1ST—MALPOSITIONS—ABERRATIONS OF LEVEL, OR NORMAL PLANE.

*Influence on Menstruation, Conception and Pregnancy.*

| | |
|---|---|
| *a.* Ascent.<br>*b.* Descent.<br>c. Prolapsus.<br>*d.* Procidentia. | Menstruation modified—generally excessive, without pain. Prospects of conception—not always, but generally, impaired. Pregnancy insecure—liable to abortion until after quickening. |

GENUS 2D. OBLIQUITIES OR DEVIATIONS.—ABERRATIONS OF DIRECTION, OR OF NORMAL AXIS.

*Influence on Menstruation, Conception and Pregnancy.*

| | |
|---|---|
| *a.* Anteversion.<br>*b.* Retroversion.<br>*c.* Latero-version. | Menstruation disturbed—generally painful.<br>Prospects of conception always seriously impaired.<br>Abortion always imminent at time of quickening. |

GENUS 3D. DISTORTIONS—ABERRATIONS OF FORM.

*Influence on Menstruation, Pregnancy and Conception.*

| | |
|---|---|
| *a.* Anteflexion.<br>*b.* Retroflexion.<br>*c.* Lateroflexion.<br>*d.* Inversion.<br>SUB-GENUS.*<br>*e.* Cervical Elongation.<br>*f.* Cervical Constriction.<br>*g.* Tubal Constriction. | In most of the species, menstruation is deficient and painful—"obstructive dysmenorrhœa"—conception almost hopeless, from non-contact of "germ-cells with sperm-cells." In rare cases of accidental conception, (as may happen to some of the species) abortion, in most of them, almost inevitable. Where the conditions of the sub-genus exist to the full degree, conception is impossible. |

As the title of the paper would indicate, position and what he styles "pneumatic pressure," has the fullest consideration in Dr. Campbell's report. He urges the importance and indispensable necessity of knee and breast posture, in the treatment of nearly all those conditions which can properly be called uterine displacements. He reports, in full, his satisfactory experience in the use of this "postural treatment" of uterine displacements, during a period of over thirty years. "It has been an *unwritten tradition* among many of the physicians of our school for years before my own time." In tracing the history of knee-and-breast posture, he finds that it is, by no means, a new or recent device—finding mention of it as applied, for one object or another, since the year 1701. Like many of the inventions of gynæcology—as the speculum, the sponge-tent and uterine sound—"genu-pectoral posture" has been the subject, time after time, of discovery, oblivion, re-discovery, neglect and revival. The most recent revival of its application was that of Dr. Marion Sims, in 1852. His recommendations of it, *for a brief period*, called general at-

*These are associated as a "sub-genus"—for, though generally described with displacements, they are only distortions, but not displacements. We might, to the genera and species here arranged, have added what might be called "varieties"—thus: In every species of displacement there are *degrees;* there are all degrees of downward displacement—the division into species is sufficient for them—but in the "Obliquities," these degrees of fundal depression might be graded into varieties; as also in the lateral deviations. Still more pertinent would be a series of varieties in the "Distortions," all degrees and directions of curve or angular bend; also the *localities* of the curve or bend; whether in the neck or in the body, (as some claim may occur) or at the junction of neck and body. Then again, in the sub-genus of distortions, we might also establish *varieties;* as the kinds and degrees of cervical elongation; the kind and degrees of cervical constriction; whether congenital or acquired, whether plastic or inflammatory, complete or incomplete, straight or devious, etc. Again, in "tubal constriction," whether single or double, inflammatory or congestive or indurated, etc. All these abnormalities might be introduced, to constitute varieties, in legitimate accordance with the true principles of classification, but our earnest desire is *to be useful*, and such additions might complicate the *tableau* to such an extent that it might not be entertained and adopted by the profession. —*Note added by Reporter in reviewing proof sheets of Resume.*—EDITOR.

tention to it, for only a particular class of operations—vesico-vaginal fistula, etc., but never secured its application by the profession as an instrumentality in replacements of any form of uterine malposition. Dr. Sims, though he understood apparently its capabilities, did not appear to value it properly *as a means of replacing the dislocated uterus.* As in retroversion, the displacement for which, above all others, it is most valuable, he places the patient in "semi-prone position," abandoning gravity and pneumatic pressure, and using sponge probangs and a complicated process to *imperfectly* restore the displaced organ.

Dr. Campbell adduced the writings of the late Dr. Milton Antony, of Augusta, as the most important revival of knee-and-breast position in recent times; also as the revival which, had it been generally published and known, would have extended its applications to a wider range of cases and to the relief of a far more frequent and common class of distresses than any of the applications it has found in its more recent inauguration by Dr. Sims. Dr. Antony's recommendation of the manœuvre was made in a series of papers published in the years 1836 and 1837; and it was for uterine displacements and examinations especially that he recommended it. Versions and flexions of the unimpregnated uterus were not, at that time, generally known; so that it was to the downward displacements alone that Milton Antony applied the method. Dr. Campbell claims that, since his earliest days of practice, he has used this postural treatment, applying it to every displacement possible, as its value became understood by him. Using it as a means of diagnosis as well as of treatment, he insists that no investigation of any form of uterine displacement can be thorough or conclusive without "genu-pectoral" posture and "pneumatic pressure," by which we judge regarding the mobility, the extent of motion and the direction of motion possible to the displaced organ. He insists that no pessary should ever be applied, or its application attempted, *until after the dislocated organ has been reduced by knee-and-breast posture, assisted by pneumatic pressure.* The ordinary introduction of the pessary generally involves a *painful pushing up* of the prolapsed or retroverted womb *upon the pessary;* whereas the womb *should first be replaced* to its fullest extent, in knee-and-breast posture, and then the pessary *laid upon* the posterior vaginal *cul-de-sac,* while the woman is in the inverted position. As the patient rises to the *erect* kneeling posture, the womb settles down upon the posterior bar, or segment of the pessary, and thus she *begins* its use with comfort and freedom from pain. The particular rules, given by Dr. Hodge, for the application of his open lever pessary are diametrically reversed by Dr. Campbell, so as to adapt them to his far superior application of this valuable instrument in the "genu-pectoral position," after full reduction by "pneumatic pressure."

SELF-REPLACEMENT.—That which may be regarded as perhaps the most triumphant achievement in the use of this most powerful instrumentality for uterine reposition is what Dr. Campbell himself has termed "Self-replacement."

*Breaking the Suction.*

In his discussion of the rationale of the "genu-pectoral position," he demonstrated clearly to all present that like a pneumatic pump (which he actually used in the demonstration,) the descended womb, resting against the closed vulva, would not return to its position, even when dragged upon by its own weight, together with all the weight of the abdominal viscera, unless, as he expressed it, "*the suction is broken,*" by the separation of the labia, to introduce air into the vagina, any more than was the piston of the reversed pneumatic syringe dragged down by the heavy book attached to the handle, until the thumb was removed from the opening above the plunger. He holds that in reduction of retroversions, except, in cases of adhesion or impaction, *nothing is necessary but "genu-pectoral pneumatic pressure,"* and further, that Simpson's repositor, Bond's repositor, Sims' probangs, and the colpeurynter are useless instruments, except in extraordinary cases.

Dr. Campbell stated that for years he had found difficulty in securing to patients the full advantages of knee-and-breast posture from the fact that the womb would not replace itself, unless air was allowed to enter the vagina. His advice of "nightly replacement," only practicable by the patient herself, but in his mind a very great desideratum, constantly failed to afford relief on this account; also in the case of patients in the condition of virginity, often the subjects of displacements, but averse to any manipulative treatment, simple "genu-pectoral" posture would not relieve. He therefore, after many progressive attempts, perfected a simple instrument to the purpose of "pneumatic reduction of the dislocated uterus."

Dr. Sims' duck-bill speculum, as used by Dr. Sims in his operations within the vagina, supplied him with an efficient and suggestive model for devising a method of establishing an "air-way" for "breaking the suction." This instrument, of course, was too expensive to supply one each to perhaps dozens of patients to whom he would wish advantageously to recommend "nightly self-replacement." He devised a curved staff of vulcanite, some eighteen inches in length, tunnelled by a *bore* from the end to the curve, directing the patient to assume the position, and to reach back and introduce the perforated curved end into the vagina, that the air might enter through the tunnel. This was found, in the first place, still too expensive, and then inconvenient, on acccount of the interference by clothing, as well as by its inherent awkwardness of application.

*Campbell's Uterine Repositor.*

His "*Pneumatic Self-Repositor*" is the idea of an "air-way" reduced to the last degree of economy, simplicity and convenience. They are so cheap that they can be ordered from the manufacturer by the gross at a price little above the cost of ounce vials, and given if desired without charge to each patient. It consists essentially of a glass tube of various forms, from two and one-half to three inches long, slightly curved near the end and bulbous, to admit of easy introduction. He presented specimens of various forms and sizes of this instrument. Some, very attenuated in calibre, to use in virgins, to pass above the hymen, which is generally more or less lunated, without injury to that important membrane. The application is very simple. The patient assumes the "genu-pectoral position," and, while thus placed, or before rising, with or without lubrication, she introduces the tube, only for a moment. The air rushes in, the suction is broken, and immediately, whatever may be the displacement, unless there is adhesion or impaction, "self-replacement" is completely and instantly accomplished. She then lies quietly down for the night, and a night's rest, with unstretched uterine ligaments, and unimpeded uterine circulation, if often and regularly repeated, will, at least, go far in favoring a restoration to a permanently normal position of the organ.

The text of the report is illustrated by a number of original engravings, from the pencil of Dr. A. Sibley Campbell, of Augusta. These represent the several displacements of the uterus, and the process of their reduction by "position and pneumatic pressure."

Dr. C. also discusses the value of pessaries and other mechanical appliances, which he is far from discrediting. He also considers many of the attendant complications of uterine displacements and their various means of relief; but as "position and pneumatic pressure" comprehended the chief peculiarities of his report, we wait the appearance of the forthcoming volume of Transactions, rather than extend any further what was intended as only a compendious resume.

Dr. Battey, of Atlanta, rose and said: The mention of the name of Milton Antony, in Dr. Campbell's report, stirs within me feelings of admiration, of gratitude and of deepest veneration. He was the friend and medical adviser of my father and of my sainted mother, who, with him, have long since gone to their rest, and hence *my* friend. His kindly and succoring hand opened for the first time my eyes to the light of glorious day, and safely conducted me through the perils of puling infancy. He died while I was yet too young to know of anything but his kindness and almost God-like benevolence. That he was a man of genius and untiring energy his works, that after his rest now do follow him, undeniably attest: The Medical College of Georgia, of which he was the founder; the Southern Medical and Surgical Journal, long the only one, and always a most valuable medium of interchange of medical knowledge in the South, was the creature of his active brain; and now the enterprise and research of our reporter revives and elaborates from its antiquated and dusty volumes this signal token that good seed sown, even by the wayside, do not always perish, but in due time may spring up and bear the richest fruit, bringing forth forty, sixty or an hundred fold!

I have often tried to avail myself, for the benefit of my patients, of the ease, comfort and relief which are attributed to the "knee-and-breast posture" by advising them to practice it. I was often disappointed; and now I am made aware that the failure in relief arose from the fact that "the suction was not broken," and because there was not at hand any convenient and efficient means of "air-way," such as we now have offered to us in Dr. Campbell's simple and most philosophical device, the "pneumatic self-repositor." X.

## Figures and Tables

**Figure f1:**
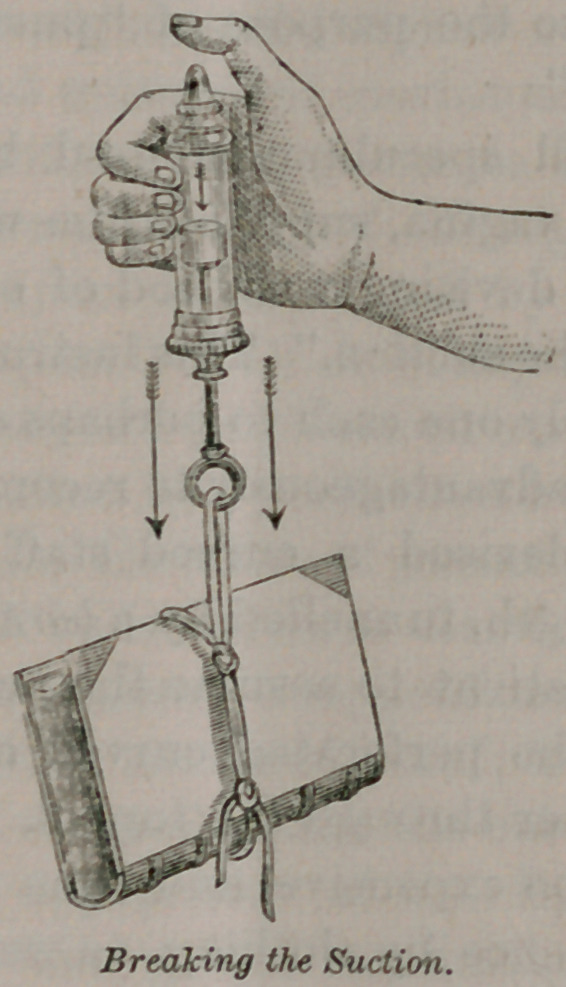


**Figure f2:**